# A Biochemical Approach to Study the Role of the Terminal Oxidases in Aerobic Respiration in *Shewanella oneidensis* MR-1

**DOI:** 10.1371/journal.pone.0086343

**Published:** 2014-01-22

**Authors:** Sébastien Le Laz, Arlette Kpebe, Marielle Bauzan, Sabrina Lignon, Marc Rousset, Myriam Brugna

**Affiliations:** 1 CNRS, Aix-Marseille Université, Laboratoire de Bioénergétique et Ingénierie des Protéines, UMR 7281, IMM, Marseille, France; 2 CNRS, Aix-Marseille Université, Unité de fermentation, FR3479, IMM, Marseille, France; 3 CNRS, Aix-Marseille Université, Plate-forme Protéomique, FR3479, IMM, MaP IBiSA, Marseille, France; National Research Council of Italy (CNR), Italy

## Abstract

The genome of the facultative anaerobic γ-proteobacterium *Shewanella oneidensis* MR-1 encodes for three terminal oxidases: a *bd*-type quinol oxidase and two heme-copper oxidases, a A-type cytochrome *c* oxidase and a *cbb*_3_-type oxidase. In this study, we used a biochemical approach and directly measured oxidase activities coupled to mass-spectrometry analysis to investigate the physiological role of the three terminal oxidases under aerobic and microaerobic conditions. Our data revealed that the *cbb*_3_-type oxidase is the major terminal oxidase under aerobic conditions while both *cbb*_3_-type and *bd*-type oxidases are involved in respiration at low-O_2_ tensions. On the contrary, the low O_2_-affinity A-type cytochrome *c* oxidase was not detected in our experimental conditions even under aerobic conditions and would therefore not be required for aerobic respiration in *S. oneidensis* MR-1. In addition, the deduced amino acid sequence suggests that the A-type cytochrome *c* oxidase is a *ccaa*_3_-type oxidase since an uncommon extra-C terminal domain contains two *c*-type heme binding motifs. The particularity of the aerobic respiratory pathway and the physiological implication of the presence of a *ccaa*_3_-type oxidase in *S. oneidensis* MR-1 are discussed.

## Introduction

In the oxygen respiratory systems, electrons of low-redox potential electron donors are transferred through a series of membrane-bound proteins or complexes and finally, the reduction of molecular oxygen to water is catalyzed by enzymes called terminal oxidases. These oxygen reductases are complicated integral membrane multi-subunit complexes grouped into two major superfamilies. Most of them belong to the well-characterized heme-copper oxidases (HCO) superfamily [1]. HCO have been named cytochrome *c* oxidases or quinol oxidases, depending on the nature of their electron donor and are able to pump protons across membrane. Additionally, based on biochemical and structural differences in their catalytic subunits and on phylogenetic analysis, a classification of HCO into three families was proposed [2]: i) type A (mitochondrial-like oxidases or *aa*_3_-type), ii) type B (*ba*_3_-type oxidases) and iii) type C (*cbb*_3_-type oxidases) only detected in bacteria [3]. Cytochrome *bd*-type oxidases, phylogenetically unrelated to the HCO [4], represent the second major oxidases superfamily [5]. Widely distributed among prokaryotes, *bd*-type oxygen reductases function as quinol-oxidases and are bioenergetically less efficient than HCO since they generate a proton motive force by transmembrane charge separation rather than by pumping protons [6]–[8]. In addition to its role in cell bioenergetics, many studies suggest that cytochrome *bd* oxidase may be implicated in other important physiological functions. In particular, the enzyme seems to be involved in the bacterial response to a wide variety of stress conditions such as alkalinization of the medium, high temperatures, hydrogen peroxide and nitrosative (NO) stresses [5], [9]–[11]. Cytochrome *bd* oxidase could also play a determinant role in bacterial pathogenicity by protecting bacteria against the NO-mediated host immune response [12].

In contrast to the mitochondrial respiratory systems, most bacteria have branched-respiratory chains terminating in multiple oxidases or use alternative electron acceptors. This enables them to respond to changes in the environment and contributes to their ability to colonize many microoxic and anoxic environments [13]–[15]. Each oxidase type in bacteria is expected to have a specific affinity for O_2_ [16]. Under O_2_ limitation, many bacteria induce high O_2_-affinity oxidases to respire traces of molecular oxygen. The *cbb*_3_-type cytochrome *c* oxidase and the *bd*-type quinol oxidase are generally considered to have a high affinity for oxygen [17], [18]. On the other hand, the A-type oxidases have low affinity for oxygen and are predicted to provide the capacity to grow in high-O_2_ environments only [16].

*Shewanella* species are facultative anaerobic gram-negative γ-proteobacteria that exhibit extensive respiratory versatility using a broad spectrum of terminal electron acceptors such as fumarate, dimethyl sulfoxide, NO_2_^−^, NO_3_^−^, Fe(III), As(V), U(VI), Mn(IV), Cr(VI), Tc(VII) elemental sulfur and azo dyes [19]–[22]. Genome analysis of *Shewanella oneidensis* MR-1 revealed the presence of genes coding for terminal oxidases: a *bd*-type quinol oxidase and two HCO, a A-type cytochrome *c* oxidase and a *cbb*_3_-type oxidase [19]. Latterly, it has been reported that cytochrome *bd* oxidase confers nitrite resistance to *S*. *oneidensis* MR-1 and plays a significant role in oxygen respiration under microaerobic but not aerobic conditions [23]. Marritt *et al*. [24] hypothesized that, in accordance with their predicted affinity for molecular oxygen, *S. oneidensis* MR-1 A-type and C-type cytochrome *c* oxidases should operate under aerobic and microaerobic conditions respectively. However, the physiological role of the specific terminal oxidases in *S. oneidensis* MR-1 remains to be revealed.

Very recently, while our work was in progress, Zhou *et al*. [25] conducted a relevant study mainly based on gene expression providing a better understanding of the oxygen respiratory metabolism by *S. oneidensis* MR-1. However, because of translational repressor proteins, antisense RNA and posttranslational modifications, mRNA abundance level can be unrelated to active protein abundance level. Thus, many studies suggest that mRNA expression patterns are necessary but insufficient for quantitative description of biological systems [26]–[29]. For example, comparison of the transcriptome and proteome data for 27 proteins regulated by the ferric uptake regulator in *S. oneidensis* MR-1 revealed that the expression patterns was correlated with the gene expression data for about half of the proteins whereas microarray data and proteome data were not correlated or even inversely correlated for the others proteins [27].

In this work, we used a biochemical approach and directly measured oxidase activities coupled to mass-spectrometry analysis to further characterize the terminal segment of the respiratory chain of *S. oneidensis* MR-1 depending on the growth conditions. Measurements performed on solubilized membranes of wild-type and terminal oxidases deficient strains clearly indicate that the quinol oxidase activity, arising from the *bd*-type oxidase, is extremely higher under microaerobic conditions than under aerobic conditions which is consistent with the fact that *bd*-type oxidases are generally induced under O_2_ limited conditions. Surprisingly, *cbb*_3_-type oxidase is the only cytochrome *c* oxidase present in *S. oneidensis* MR-1 membranes under both aerobic and microaerobic conditions. The overall results suggest that the A-type cytochrome *c* oxidase, predicted to have a low affinity for O_2_, is not present in the bacterium, in our growth conditions, whatever the oxygen tension. A thorough comparison between our results and those obtained by Zhou and colleagues [25] is developed in the discussion section.

## Experimental Procedures

### Bacterial Growth Conditions

The bacterial strains used in this study are listed in Table 1. All the strains were maintained on Luria-Bertani (LB) agar plates containing the appropriate antibiotic. Overnight cultures of the *Escherichia coli* strains CC118 λpir and 1047/pRK2013 used for conjugations were carried out in LB medium at 37°C and 250 rpm. *S. oneidensis* MR-1 and terminal oxidase-deficient strains were grown in LB medium at 30°C either in aerobic conditions or microaerobic conditions. A 30-L BIOSTAT® Cplus-C30-3 fermentor (Sartorius BBI Systems, Germany), controlled by a micro-DCU system and equipped with pH and pO_2_ sensors, was used for 30-L batch cultures. Bioreactor was inoculated at OD600 nm∼0.2 with 1 L of an overnight flask culture of *S. oneidensis* MR-1 in LB. The pH of the LB medium was regulated at 7.2 with 5 M H_3_PO_4_. The oxygen partial pressure sensor (InPro® 6820, Mettler-Toledo, Switzerland) was calibrated at 100% in air-saturated LB at 30°C which corresponds to an estimated oxygen concentration of 8 mg.L^−1^ (250 µM). For aerobic cultures, the pO_2_ was regulated at a minimum of 40% (100 µM O_2_) whereas for microaerobic cultures the pO_2_ was regulated at a maximum of 6.5% (16 µM O_2_). During growth, samples were taken from the cultures at the exponential phase and at the stationary phase, cells were harvested 10 min at 11,400×g and pellets were frozen at −80°C. For growth parameters measurements, strains were inoculated at a OD600 nm∼0.05 in 500 mL-flask with baffled base containing 100 mL LB and were aerobically grown at 30°C and 250 rpm. Samples were periodically taken in sterile conditions.

**Table 1:** Strains used in this study.

Strain	Description	Reference or source
***E. coli***		
DH5α	Competent cells for cloning	New England Biolabs
1047/pRK2013	Helper stain for conjugation	[76]
CC118λpir	Host strain for pKNG101 replication and donor strain for conjugation	[77]
***S. oneidensis*** a		
MR-1	Wild-type	Laboratory collection
SLL01	SO2364 deletion mutant/Δ*ccoN*	This study
SLL02	SO3286 deletion mutant/Δ*cydA*	This study
SLL03	SO4607 deletion mutant/Δ*coxA*	This study
SLL05	SO4607 and SO3286 deletion mutant/Δ*coxA*Δ*cydA*	This study
SLL06	SO4607 and SO2364 deletion mutant/Δ*coxA*Δ*ccoN*	This study

a All *S. oneidensis* strains derived from the parental strain *S. oneidensis* MR-1.

### Construction of the Deletion Mutants

*S. oneidensis* deletion mutants were constructed by double crossover events using the suicide plasmid pKNG101 as previously described [30]. Briefly, 500 bp regions upstream and downstream the gene to be deleted were amplified by overlapping PCR with Pfu DNA polymerase (Promega) and *S. oneidensis* MR-1 genomic DNA as template. Primers used for generating PCR products are listed in Table S1. The purified PCR product was cloned into the pGEM®-T Easy vector (Promega) and subcloned into pKNG101 using restriction sites SpeI and SalI. The resulting plasmid was transferred into the plasmid donor strain *E. coli* CC118λpir and mobilized into the appropriate *S. oneidensis* strain by conjugation with the helper strain *E. coli* 1047/pRK2013. Integration of the mutagenesis construct into the chromosome was selected by streptomycin resistance. Clones in which the double recombination events occurred were selected on LB agar plates containing 6% sucrose. Colonies that were resistant to sucrose and susceptible to streptomycin were screened for the deletion event by PCR and confirmed by DNA sequencing. Following this strategy, the SO2364, SO4607 and SO3286 genes encoding respectively the catalytic subunit of the annotated *cbb*_3_-type cytochrome *c* oxidase, cytochrome *c* oxidase and cytochrome *d* ubiquinol oxidase were deleted. All the single and double deletion strains are listed in Table 1.

### Preparation and Solubilization of Membranes

About 2 g of cells were resuspended in 20 mM Tris-HCl pH 7.6 supplemented with DNase I and protease inhibitors (Roche) and disrupted twice with an ultra high pressure cell homogenizer (Stansted Fluid Power). Debris and unbroken cells were removed by centrifugation for 10 min at 5,000×g and the supernatant was ultracentrifuged for 1 h at 230,000×g at 4°C to pellet the membranes. Membranes were gently resuspended in 20 mM Tris-HCl pH 7.6 with 5% glycerol to a protein concentration of 10 mg.mL^−1^ and *n*-dodecyl β-D-maltoside (DDM) was added to a final concentration of 1% (w/v). Solubilization was performed on a rotary tube mixer for 2 h at 4°C. After ultracentrifugation for 1 h at 230,000x g at 4°C, the supernatant containing the DDM-solubilized membranes was retained and protein concentration was determined with a bicinchoninic acid protein assay kit (Sigma) using bovine serum albumin as protein standard.

### Spectral Analysis on Solubilized Membranes

Reduced minus oxidized difference absorbance spectra were recorded on a Lambda 25 UV/VIS spectrophotometer (PerkinElmer) at room temperature. 1 mM potassium ferricyanide was used as the oxidizing agent and a few grains of sodium dithionite was used as the reducing agent.

### Cytochrome *c* Oxidase Activity and O_2_ Uptake Measurements

Cytochrome *c* from equine heart was reduced with 75 mM sodium ascorbate in 50 mM sodium phosphate buffer pH 7 with 5% glycerol (v/v). Excess of reductant was removed using a PD10 column (GE Healthcare) and reduced cytochrome *c* was stored at −20°C. Cytochrome *c* oxidase activity was spectrophotometrically measured, on a Lambda 25 UV/VIS spectrophotometer (PerkinElmer) at 550 nm at 30°C in a reaction mixture containing 50 mM sodium phosphate buffer pH 7.4 and 75 µM reduced cytochrome *c*. The reaction was initiated by the addition of solubilized membranes. O_2_ reductase activity on solubilized membranes was polarographically measured with a Clark-type oxygen electrode (Hansatech Instruments Oxygraph) in a stirred volume of 1 mL of 50 mM sodium phosphate buffer pH 7.4 using either ubiquinol-1 or *N*,*N*,*N*′,*N*′-tetramethyl-*p*-phenylenediamine (TMPD) as electron donor. Ubiquinol-1 was prepared from ubiquinone-1 (Sigma) by reduction with sodium dithionite and sodium borohydride as previously described [31]. Quinol oxidase activity was determined in the presence of 0.9 mM ubiquinol-1 and 10 mM DTT whereas TMPD oxidase activity assays were carried out with 100 µM TMPD and 10 mM sodium ascorbate. Reactions were initiated by the addition of DDM-solubilized membranes and the consumption of dioxygen was recorded at 30°C. Data were analyzed using the O_2_ View software (version 1.02; Hansatech Instruments Ltd.). For inhibition studies, membranes were pre-incubated with 50 µM KCN for 10 min and added to the reaction mixture containing 50 µM KCN.

### Separation by Blue-native gel (BN-gel) Electrophoresis

BN-gels were performed using a mini VE cell electrophoresis apparatus (Amersham Pharmacia) according to the method of Schägger [32], [33], as described by Guiral *et al*. [34] except that the intensity was 10 mA/gel and the voltage 250 V. Running times were 3 h. The gels were run with the blue cathode buffer (50 mM Tricine, 7.5 mM imidazole pH 7.0, 0.02% Coomassie Blue G 250 (Serva Blue G)) until the front line had entered into half of the gels and then the cathode buffer was replaced by the one that contained only 0.002% Serva Blue G. The anode buffer was constituted of 25 mM imidazole pH 7.0. The apparent molecular mass of proteins was estimated using the native molecular mass markers NativeMark (Invitrogen).

### In-gel Enzymatic Activities

Cytochrome *c* oxidase activity was revealed on the BN-gel at 30°C using 3,3′-diaminobenzidine and cytochrome *c* from equine heart as previously described [34]. TMPD oxidase activity was revealed on the BN-gel at 30°C using 1 mM TMPD as electron donor in 50 mM sodium phosphate buffer pH 7. Protein bands were cut out from the gel and stored at −20°C before mass spectrometry analysis.

### Protein Identification by In-gel Digestion and Mass Spectrometry

Tryptic digestion experiments: a robotic workstation (Freedom EVO 100, TECAN) was used to perform automated sample preparation, including multiple steps: washes, reduction and alkylation, digestion by trypsin (PROMEGA, proteomics grade with 0.025% ProteasMax) and extraction of tryptic peptides.

Electrospray quadrupole time of flight (ESI-Q-ToF) analyses were performed on a Synapt G1 mass spectrometer (Waters, Manchester) equipped with a NanoLockSpray ion source and coupled to a nano flow UPLC nanoAcquity (Waters, Manchester). Tryptic peptides were dissolved in 3% acetonitrile/0.1% TFA in water and desalted on a C18 nano trap, before on line elution onto a C18 column (BEH 130 C18, 100 µm x 10 cm, 1.7 µm, Waters). Peptides were eluted with a linear gradient from 3% to 50% of mobile phase B (100% acetonitrile/0.1% formic acid) in A (0.1% formic acid in water) for 30 min. The peptides were detected into the mass spectrometer in a positive ion mode using the MS^e^ mode. The doubly charged ion of GluFibrinopeptide (785,84 Da) was used as lock mass. Processing of the spectra and protein search were made by Protein Lynx Global Server 2.5.2 (Waters) using the following parameters: *Shewanella oneidensis* database (8 674 entries, with keratins and trypsin), trypsin enzyme, two miscleavages, carbamidomethylation of cysteine as a fixed modification, oxidation of methionine as an optional modification and a mass tolerance set as automatic. Proteins were considered as identified by passing these two filters: minimum 5 fragment consecutive ions from the b/y series matches per peptide and minimum 2 peptide matches per protein.

### Sequence Analysis

Membrane-spanning helices were searched for using the program TMpred [35]. Multiples sequences were aligned using the program CLUSTAL W [36] and the HCO phylogenetic tree including various *Shewanella* A-type cytochrome *c* oxidases was built with MEGA5 (http://www.megasoftware.net) [37].

## Results

### Sequence Analysis of the *S. oneidensis* MR-1 Terminal Oxidases

#### The *cbb*_3_-type oxidase (Fig. 1A)

The structural subunits of the *cbb*_3_-oxidase are encoded by four genes (SO2364-SO2361) organized in the putative *ccoNOQP* operon. Sequence analysis of *ccoN,* encoding the catalytic subunit of 53.5 kDa, indicates 12 predicted transmembrane helices which harbor the conserved histidines required for ligating the heme *b* and the heme *b*_3_/Cu_B_ binuclear center [38]. Inspection of the derived amino acid sequences revealed that *ccoO* and *ccoP* encode a monoheme *c*-type cytochrome containing a predicted N-terminal transmembrane helix and a diheme *c*-type cytochrome containing two predicted N-terminal helices respectively. *ccoQ* is predicted to encode a small protein of 6.5 kDa containing a transmembrane helix. The *ccoGHIS* (or *fixGHIS*) gene cluster whose expression is required for the assembly of a functional *cbb*_3_ oxidase is found close to the *ccoNOPQ* operon in most bacteria expressing a *cbb*_3_-type oxidase [39]–[41]. Interestingly, *ccoG,* encoding a putative polyferredoxin, is a monocistronic predicted gene (SO4737), not genomically adjacent to the *cco* genes. Moreover, an additional gene annotated disulfide bond oxidoreductase *dsbD* (SO2357), is found downstream of *ccoS*. Its predicted amino acid sequence exhibits significant similarity (77%) with the protein VC1435 from *Vibrio cholerae* located at the same position in the *cco* gene cluster and required for assembly of the *cbb*_3_ oxidase [42].

**Figure 1:**
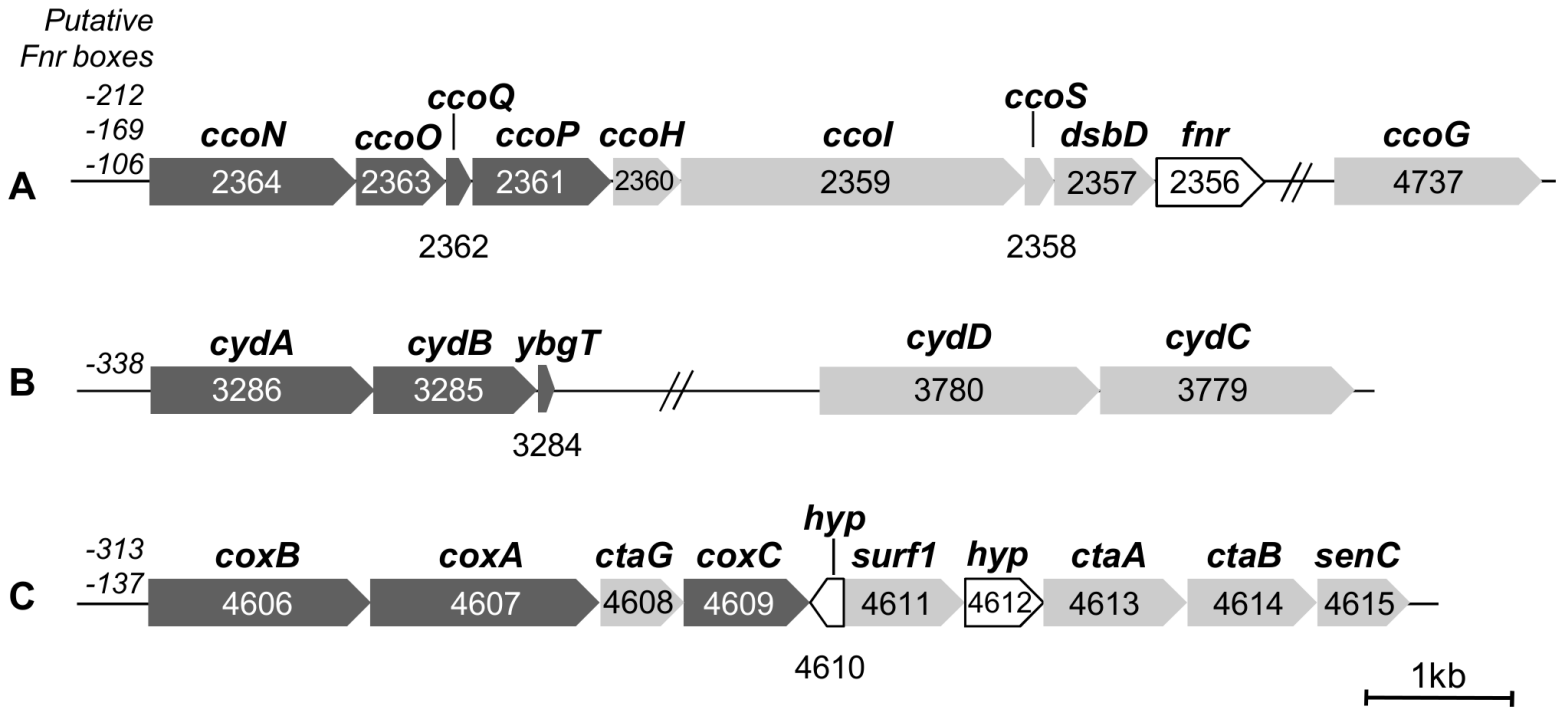
Gene clusters encoding the respiratory oxidases of *S. oneidensis* MR-1. Genes encoding the structural subunits are shown in dark grey, genes involved in maturation and assembly of the oxidases are shown in light grey and genes encoding hypothetical proteins (*hyp*) or other proteins are shown in white. Upstream each gene cluster are indicated the positions of the putative Fnr boxes. The number indicated for each gene corresponds to the locus tag. Gene clusters encode the *cbb*_3_ oxidase (A), the *bd*-type quinol oxidase (B) and the A-type cytochrome *c* oxidase Cox (C). The scale bar represents 1 kb.

#### The *bd*-type quinol oxidase (Fig. 1B)

The gene cluster SO3286–SO3284 comprises the genes coding for the two subunits CydA (58 kDa) and CydB (42 kDa). The deduced amino acid sequences reveal a high similarity to CydA and CydB from *E. coli* (71% and 66.5% of sequence identity, respectively). CydA is predicted to have nine transmembrane helices and sequence alignment clearly indicates that the residues ligating the low-spin heme *b*_558_ and the high-spin heme *b*_595_ are conserved in *S. oneidensis* MR-1. CydB is predicted to contain eight membrane-spanning helices like its counterpart in *E. coli* [5]. A third gene located in the 3′ region of *cydAB* and annotated *ybgT* in the *S. oneidensis* MR-1 genome encodes a very small protein of 38 aa predicted to contain a membrane spanning helix. This protein shares 58% sequence identity with the *E. coli* YbgT homologue designated as CydX which has recently been described as a subunit of the cytochrome *bd* oxidase complex required for complex activity [43]. A second gene cluster (SO3780–SO3779) is composed of *cydC* and *cydD,* two genes essential for the assembly of cytochrome *bd*-I in *E. coli* [44]–[47].

#### The A-type cytochrome *c* oxidase (Fig. 1C)

The *cox* gene cluster (SO4606–SO4609) is predicted to encode the three subunits of a third terminal oxidase (Cox) annotated *aa*_3_-type cytochrome *c* oxidase in the *S. oneidensis* MR-1 genome. The predicted subunit CoxA (59 kDa) comprises 12 transmembrane helices and presents 76% of sequence similarity with the catalytic subunit of the *aa*_3_ oxidase from *Paracoccus denitrificans*. The six histidines binding the heme-copper binuclear center and the low-spin heme are conserved. The conserved residues forming the D and K-channels and the GHPE260V motif allow to classify Cox in A-type oxidases in the A1 subfamily. The predicted subunit II CoxB (55 kDa) contains the five conserved ligands of the binuclear Cu_A_ center typical of cytochrome *c* oxidase. Sequence alignment with the subunit II of the *aa*_3_ cytochrome *c* oxidase from *P. denitrificans* revealed an extension of about 230 amino acid residues in the C-terminal part of CoxB containing two *c*-type heme binding consensus sequences. This extra domain likely binds two *c*-type hemes, an uncommon feature among HCO. The predicted subunit III CoxC (37 kDa) comprises seven predicted transmembrane helices and does not contain any cofactor.

The *cox* gene cluster includes genes involved in the maturation and assembly of the oxidase. SenC is known to bind copper and to be involved in the cytochrome *c* oxidase assembly in yeast or bacteria [48]–[50]. *ctaA* (SO4613) and *ctaB* (SO4614) encode putative heme *a* and heme *o* synthase respectively. Surf1 is involved in heme *a* incorporation during bacterial cytochrome *c* oxidase biogenesis [51] and CtaG is generally considered as the assembly factor that inserts copper into the Cu_B_ center [52], [53].

As Cox from *S. oneidensis* MR-1 has never been biochemically or spectroscopically investigated, the nature of its heme cofactors is unknown. In subunit I of cytochrome *c* oxidase from *P. denitrificans*, Arginine 54 is in interaction with the formyl group of heme *a* [54], [55]. This residue which is not present in oxidases known to incorporate a different type of heme at the “low-spin site” [2] is conserved in *S. oneidensis* MR-1 CoxA. The presence of this residue as well as the *ctaA* and *ctaB* genes required for the heme *a* synthesis in the *cox* gene cluster lead us to believe that an *a*-type heme is incorporated at the “low-spin site” in the subunit I of Cox.

Finally, the presence of the *fnr* (fumarate nitrate regulator) gene directly downstream of the *cco* genes and the presence of predicted Fnr-binding sites in the upstream promoter regions of the different gene clusters suggest a direct regulation by the Fnr transcriptional regulator.

### Spectral Properties of *S. oneidensis* MR-1 Solubilized Membranes

**Figure 2:**
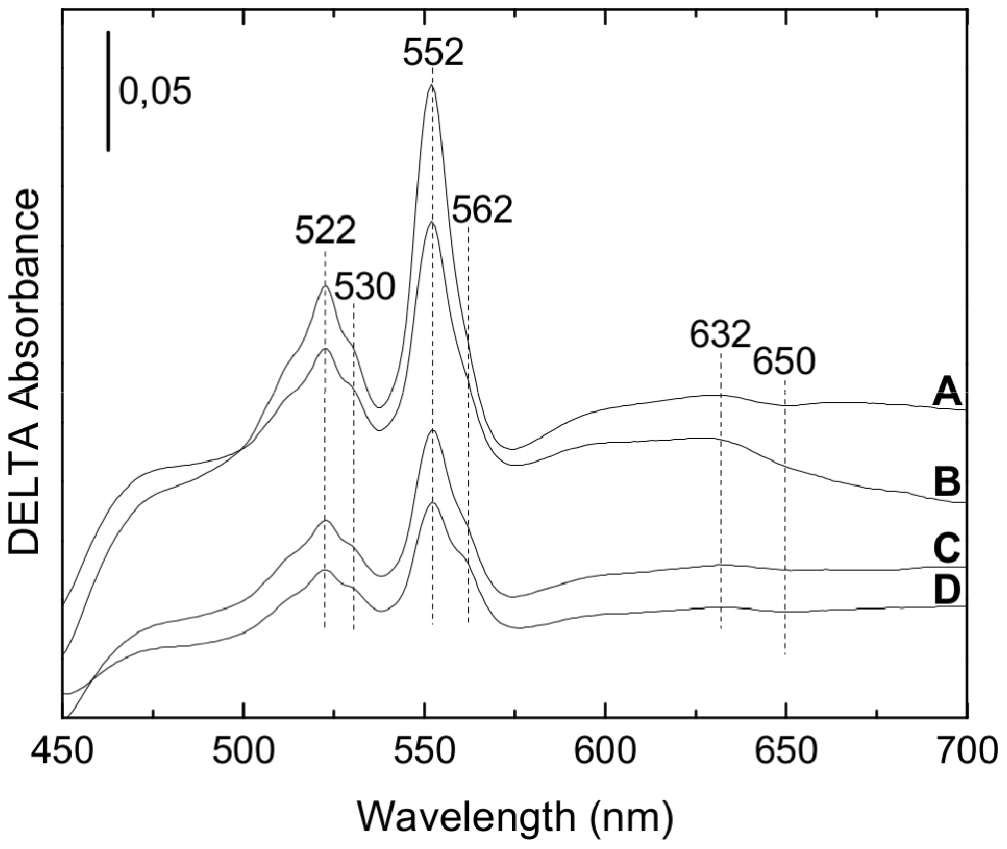
Reduced minus oxidized difference absorbance spectra of *S. oneidensis* MR-1 solubilized membranes. Solubilized membranes in 20-HCl pH 7.6, 5% glycerol, 1% DDM were oxidized using 1 mM potassium ferricyanide and reduced by adding few grains of sodium dithionite. Membranes were isolated from strain MR-1 grown under microaerobic conditions (spectra A and B) or aerobic conditions (spectra C and D). Bacterial cells were harvested in the exponential (spectra A and C) or stationary phase (spectra B and D) of growth. The concentration of proteins was 4.8 mg.mL^−1^. The spectra were recorded at room temperature. The vertical bar indicates the absorption scale.

To detect the different terminal oxidases, solubilized membranes from *S. oneidensis* MR-1 aerobically or microaerobically grown were studied by light absorption spectroscopy (Figure 2). Spectra show the presence of cytochrome *c* with peaks at 522 nm and 552 nm and cytochrome *b* with characteristic shoulders at 530 nm and 562 nm. These peaks are due to the contribution from diverse membrane cytochromes. The heme *c*/heme *b* ratio is higher (about twofold) in membranes isolated from cells grown under microaerobic conditions (Figure 2 A, B) than under aerobic conditions (Figure 2 C, D). The peak at 632 nm indicates the presence of a heme *d*, cofactor of the *bd*-type quinol oxidase and the trough around 650 nm most likely originates from a stable oxygenated cytochrome *d* species [56]. Furthermore, no *a*-type heme was detected on the spectra of membranes isolated from bacterial cells grown in microaerobic or aerobic conditions. Indeed, membranes isolated from this strain show no signal in the 440 nm-region (data not shown) and in the 600 nm-region. These results indicate the presence of the *bd*-type quinol oxidase in the bacterial membranes and the possible presence of the *cbb*_3_ oxidase in all tested conditions. However, the fact that we did not spectroscopically detect heme *a* leads us to believe that Cox is not present in *S. oneidensis* MR-1 membranes even in aerobic conditions. Nevertheless, we cannot rule out the possibility that Cox does not contain any heme *a* as a cofactor. The possible lack of the A-type cytochrome *c* oxidase in the membranes must be confirmed with other techniques.

### TMPD, Cytochrome *c* and Quinol Oxidase Activities in Solubilized Membranes of *S. oneidensis* MR-1

To further investigate the role of the different terminal oxidases in aerobic respiration in *S. oneidensis* MR-1, catalytic activity of cytochrome *c* oxidases and *bd*-type quinol oxidase were spectrophotometrically and polarographically measured with solubilized membranes prepared from cells grown in LB medium under aerobic and microaerobic conditions (Table 2).

**Table 2:** TMPD, cytochrome *c* and quinol oxidase activities in solubilized membranes of *S. oneidensis* MR-1.

	Aerobic conditions		Microaerobic conditions	
	EP^a^	SP^b^	EP	SP
TMPD oxidase activity (nmol O_2_.min^−1^.mg protein^−1^)	90+/−6	95+/−6	94+/−4	84+/−3
Cyt *c* oxidase activity (nmol cyt *c*.min^−1^.mg protein^−1^)^c^	6.2+/−0.2	8.6+/−0.5	7.3+/−0.5	5.2+/−0.6
Ubiquinol-1 oxidase activity (nmol O_2_.min^−1^.mg protein^−1^)	9+/−1	20+/−1	364+/−18	456+/−17

a EP: exponential phase of growth.

b SP: stationary phase of growth.

c cyt: cytochrome.

Listed values are averages of at least three separate experiments.

Cytochrome *c* oxidase activity was assessed by following oxidation of horse heart reduced cytochrome *c* by spectrophotometry and by monitoring O_2_ consumption in the presence of TMPD, an artificial electron donor capable of reducing cytochrome *c*. The values obtained by both methods indicate that cytochrome *c* oxidase activity is relatively constant, regardless of the tested culture conditions and growth phase.

Quinol oxidase activity of the cytochrome *bd*-type oxidase was determined by measuring O_2_ uptake with ubiquinol-1 as the electron donor. While the values obtained with membranes from cells grown in aerobic conditions were low and near to the detection limit, quinol-dependent O_2_ consumption was significantly higher with membranes from microaerobic cultures with a 20–40 fold increase depending on the growth phase. In addition, the oxygen consumption in the presence of ubiquinol-1 was lower with membranes prepared from cells harvested during the exponential phase of growth compared to the stationary phase under both aerobic and microaerobic conditions. Our data are consistent with the fact that *bd*-type oxygen reductases are generally expressed under O_2_-limited conditions i.e. in microaerobic conditions or when cultures enter the stationary phase, the decrease in oxygen tension resulting from the increase in cell density [14], [57].

Furthermore, the addition of 50 µM KCN strongly inhibited TMPD-dependent O_2_ consumption and cytochrome *c* oxidase activity (90–100%) whereas the quinol-dependent O_2_ uptake was slightly affected with a 10–15% inhibition (data not shown). This observation is in accord with the fact that a specific feature of cytochrome *bd*-type oxidase is its much lower sensitivity to cyanide than heme-copper oxygen reductases [5].

### Identification of Cytochrome *c* Oxidases by Mass Spectrometry

**Figure 3:**
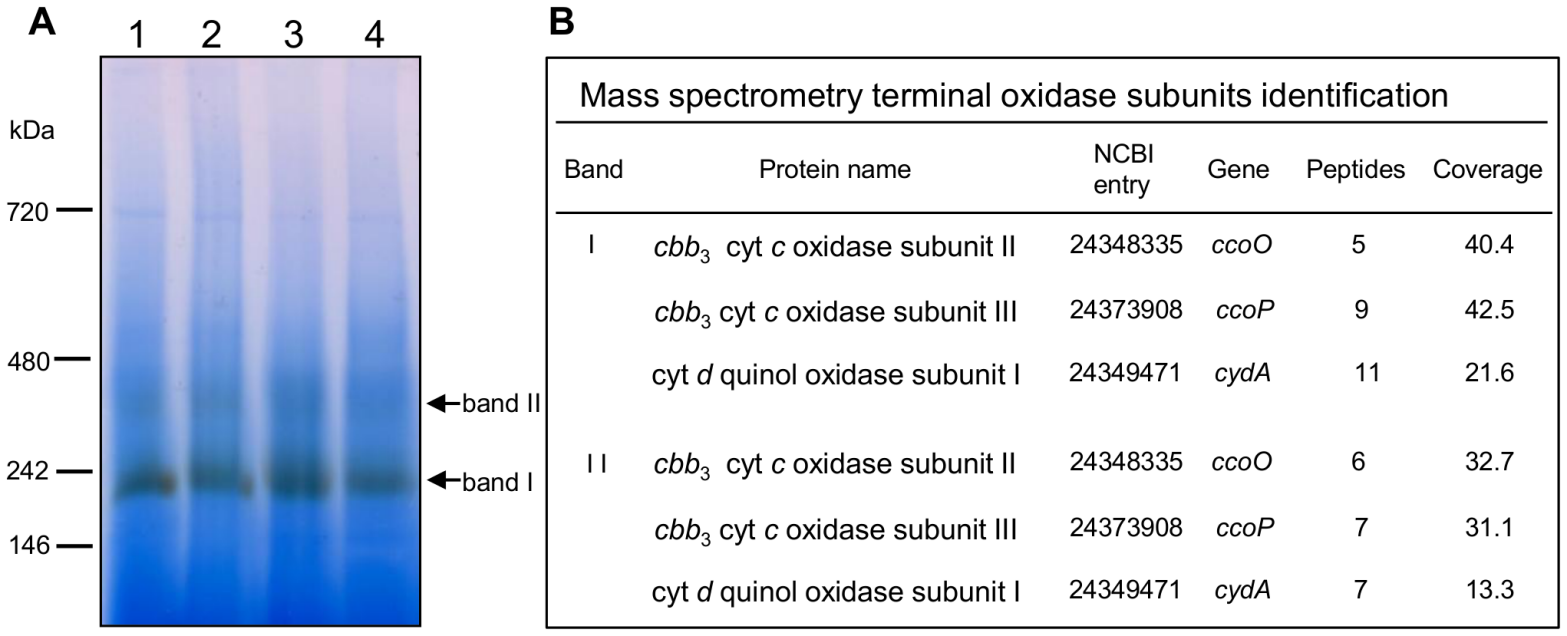
Identification of cytochrome *c* oxidase(s) in *S. oneidensis* MR-1 membranes under aerobic and microaerobic conditions. A. In-gel detection of cytochrome *c* oxidase activity in solubilized membranes on BN-gel. Membrane proteins were prepared from *S. oneidensis* MR-1 cells grown aerobically (lanes 1 and 2) or microaerobically (lanes 3 and 4) and harvested during the exponential (lanes 1 and 3) or the stationary (lanes 2 and 4) phase of growth. 130 µg of total proteins were loaded on a 5–15% polyacrylamide gel. The major brown band (band I) and the faint brown band (band II) of activity are indicated by arrows. B. Terminal oxidases subunits identified by ESI-Q-ToF mass spectrometry. Data correspond to identified proteins in bands I and II from exponentially grown cells under aerobic conditions (panel A, lane 1). Table heading: Band, roman figures refer to the protein bands from the BN gel shown in panel A; Protein name, name in NCBI database; NCBI entry, accession number; Peptides, number of unique peptides detected; Coverage, protein sequence coverage by the matching peptides (in %). Cyt: cytochrome.

In order to identify enzymes responsible for the measured cytochrome *c* oxidase activity, solubilized *S. oneidensis* MR-1 membrane proteins were separated on a BN-gel electrophoresis followed by an in-gel detection of the cytochrome *c* oxidase activity (Fig. 3A). For this purpose, *S. oneidensis* MR-1 cells were grown aerobically or microaerobically and harvested during the exponential or the stationary phase of growth to determine the influence of the oxygen level and the growth phase on the respiratory cytochrome *c* oxidase content in the membranes. BN-gel of the solubilized membranes shows one major brown band of cytochrome *c* oxidase activity, of molecular mass around 240 kDa (Fig. 3A, band I). Another faint band of activity could be detected, of molecular mass around 450 kDa (Fig. 3A, band II). The pattern of cytochrome *c* oxidase activity bands was identical whatever the oxygen tension in the medium and the growth phase. When TMPD was used instead of horse heart cytochrome *c* to reveal cytochrome *c* oxidase activity, the same pattern was also observed (data not shown). With both electron donors, no band was revealed when the in-gel detection was performed in the presence of 50 µM potassium cyanide (data not shown).

To identify the cytochrome *c* oxidases, the protein band I and band II were directly cut out from the gel, for the four growth conditions, digested with trypsin and analyzed by ESI-Q-ToF mass spectrometry (Fig. 3B). Band I and band II were found to contain, among others proteins, CcoO and CcoP, the monohemic and dihemic cytochrome *c* subunits of the *cbb*_3_ oxidase respectively. In addition, the CydA subunit of the *bd*-type quinol oxidase was also identified in band I and band II indicating that the *cbb*_3_ oxidase and the *bd*-type quinol oxidase co-migrate in the BN gel. However, none of the three subunits of the Cox enzyme was identified in solubilized membranes of *S. oneidensis* MR-1 grown microaerobically or aerobically. These results strongly suggest that, against all expectation, the high O_2_-affinity *cbb*_3_ oxidase is responsible for the cytochrome *c* oxidase activity measured in the membranes of microaerobically but also aerobically grown *S. oneidensis* MR-1 whereas the A-type cytochrome *c* oxidase Cox remains undetectable even under aerobic conditions.

### Growth Parameters and TMPD Oxidase Activity of *S. oneidensis* MR-1 Wild Type and Oxidase Deletion Mutants

The first part of the study suggests that the *bd*-type and the *cbb*_3_-type high-affinity oxidases are important under aerobic conditions while the A-type cytochrome *c* oxidase would be surprisingly dispensable despite its low affinity for O_2_. To confirm these data and determine the physiological significance of the different oxygen reductases under aerobic conditions, deletion mutants were constructed and characterized during the exponential phase of growth under aerobic conditions by measuring growth parameters and TMPD oxidase activities (Table 3). While the strain lacking the *cbb*_3_-type terminal oxidase (SLL01) grew significantly more slowly and reached the stationary phase at lower cell density compared to the wild type, the single oxidase mutants lacking the *bd*-type terminal oxidase (SLL02) or the A-type cytochrome *c* oxidase Cox (SLL03) grew as well as the wild type. Firstly, the results indicate that none of the terminal oxidases are essential for aerobic growth in our conditions. Secondly, the reduced growth rate of SLL01 suggests that the *cbb*_3_-type terminal oxidase is the predominant oxidase in exponentially growing *S. oneidensis* MR-1 cells under aerobic conditions.

**Table 3:** Growth parameters and TMPD-dependent oxidase activity of oxidase deletion mutants cultivated in aerobic conditions.

Strain	Relevant genotype	Doubling time^a^	Relative yield^b^	TMPD oxidase activity^c^
MR-1	wild-type	54.2+/−0.2	1.00	90+/−6
SLL01	Δ*ccoN*	78.0+/−0.7	0.77	0
SLL02	Δ*cydA*	53.4+/−2.1	1.00	91+/−9
SLL03	Δ*coxA*	59.0+/−1.1	0.96	97+/−6
SLL05	Δ*cydA*Δ*coxA*	56.5+/−2.7	0.97	101+/−9
SLL06	Δ*ccoN*Δ*coxA*	79.9+/−1.2	0.78	0

a Expressed in min.

b Relative yield is defined as the highest optical density in early stationary phase in mutant strains cultures relative to that in the wild-type strain cultures.

c Expressed in nmol O_2_.min^−1^.mg protein^−1^.

Data were obtained from triplicate separated experiments.

The strains lacking two terminal oxidases were also characterized. Growth parameters of the strain containing only the *cbb*_3_-type oxidase (SLL05) did not really differ from those of the wild-type indicating that the *cbb*_3_-type oxidase is sufficient to support maximum growth rate in aerobic conditions. Conversely, doubling time of the strain containing only the *bd*-type oxidase (SLL06) was significantly longer and the growth yield lower than those of the wild-type strain, with values similar to those of the single oxidase mutants lacking *cbb*_3_-type oxidase (SLL01). Interestingly, it was not possible, despite numerous efforts, to generate a double oxidase mutant lacking both *bd*-type and *cbb*_3_-type oxidases suggesting that this mutant was not viable under aerobic conditions.

Taken together, these results demonstrate that the *cbb*_3_-type or the *bd*-type oxidase is required for aerobic growth of *S. oneidensis* MR-1 and that the *cbb*_3_-type terminal oxidase is the major oxidase in exponentially growing cells. These findings are consistent with the results obtained in this work with the wild type and also suggest that the low O_2_-affinity cytochrome *c* oxidase Cox is unexpectedly not involved in aerobic respiration in our experimental conditions.

Furthermore, TMPD-dependent oxidase activity was determined with *S. oneidensis* MR-1 wild type strain and oxidase deletion mutants cultivated under aerobic conditions. First, the strain SLL06 exhibited no activity indicating that TMPD was not oxidized by the *bd*-type oxidase. This result contrasts with the cytochrome *bd* oxidase from *E. coli* which can oxidize TMPD with a quite high turnover number [58]. However, several previously reported cytochrome *bd*-oxidases have been found to exhibit low TMPD oxidase activity as in *Azotobacter vinelandii* and *Geobacillus thermodenitrificans* K1041 (formerly *Bacillus stearothermophilus* K1041), or no TMPD oxidase activity as in *Bacillus firmus* OF4 [59]–[62]. Besides, TMPD oxidase activity was not affected in the single oxidase mutants lacking *cydA* (SLL02) or *coxA* (SLL03) compared to the wild-type strain whereas no activity was measured with the strain lacking *ccoN* (SLL01). Finally, TMPD oxidase activity did not differ from that of the wild type in the strain containing only the *cbb*_3_-type oxidase (SLL05). These data indicate that TMPD oxidase activity arose from the *cbb*_3_-type oxidase only suggesting that the *cbb*_3_-type oxidase is the only HCO present in the aerobic membranes of *S. oneidensis* MR-1. Moreover, this could also explain the low values obtained from measurements of cytochrome *c* oxidase activity in Table 2 since a previous study revealed that horse heart cytochrome *c* was a poor substrate for the *cbb*_3_-type oxidase in *Vibrio cholerae,* with a rate of oxygen reduction 10–20 fold lower than with the diheme cytochrome *c*_4_ identified as a natural electron donor [63].

### Spectral Properties of *S. oneidensis* MR-1 Oxidase Deletion Mutants Solubilized Membranes

**Figure 4:**
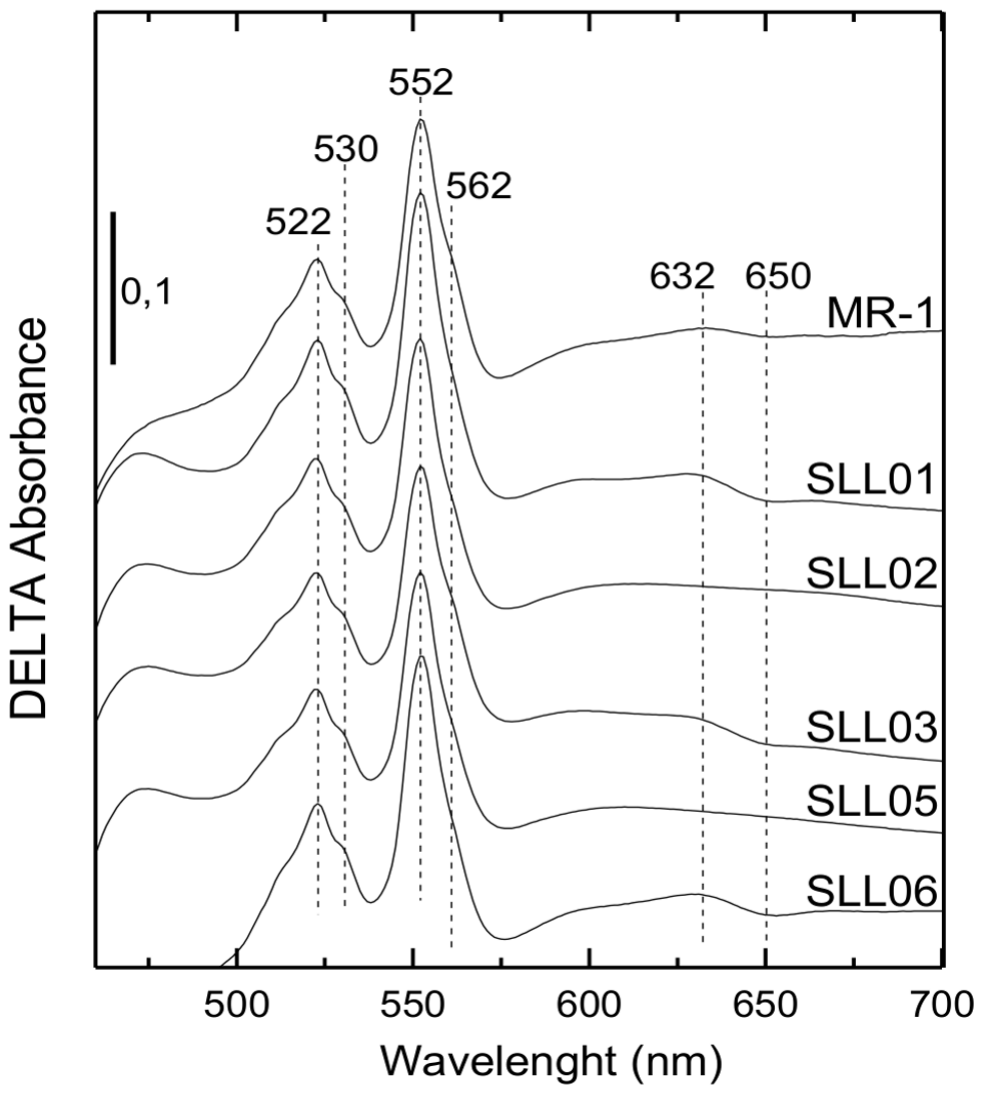
Reduced minus oxidized difference absorbance spectra 0 of membranes from aerobically grown *S. oneidensis* MR-1 oxidase mutants. Solubilized membranes in 20-HCl pH 7.6, 5% glycerol, 1% DDM were oxidized using 1 mM potassium ferricyanide and reduced by adding few grains of sodium dithionite. Membranes were isolated from aerobically grown cells harvested in the exponential phase of growth. The concentration of proteins was 11 mg.mL^−1^. The spectra were recorded at room temperature. The vertical bar indicates the absorption scale.

Solubilized membranes from aerobically grown *S. oneidensis* MR-1 and strains lacking terminal oxidases (single and double mutants) were studied by light absorption spectroscopy (Fig. 4). Dithionite-reduced minus ferricyanide-oxidized spectra demonstrate the presence of *c*-type and *b*-type cytochromes (peaks at 522, 552, and shoulders at 530 and 562 nm respectively) in membranes from all strains. The study of the mutants confirmed that the peak at 632 nm and the trough at 650 nm arise from the heme *d* of the *bd*-quinol oxidase since they are absent on spectra from SLL02 and SLL05, both strains lacking this quinol oxidase. Additionally, spectral signals for *a*-type heme (around 440 and 600 nm) were not detected in any of the strains confirming the absence of Cox in the membrane of *S. oneidensis* MR-1 in aerobic conditions.

### Identification of Cytochrome *c* Oxidases in *S. oneidensis* MR-1 Oxidase Mutants Membranes by Mass Spectrometry

**Figure 5:**
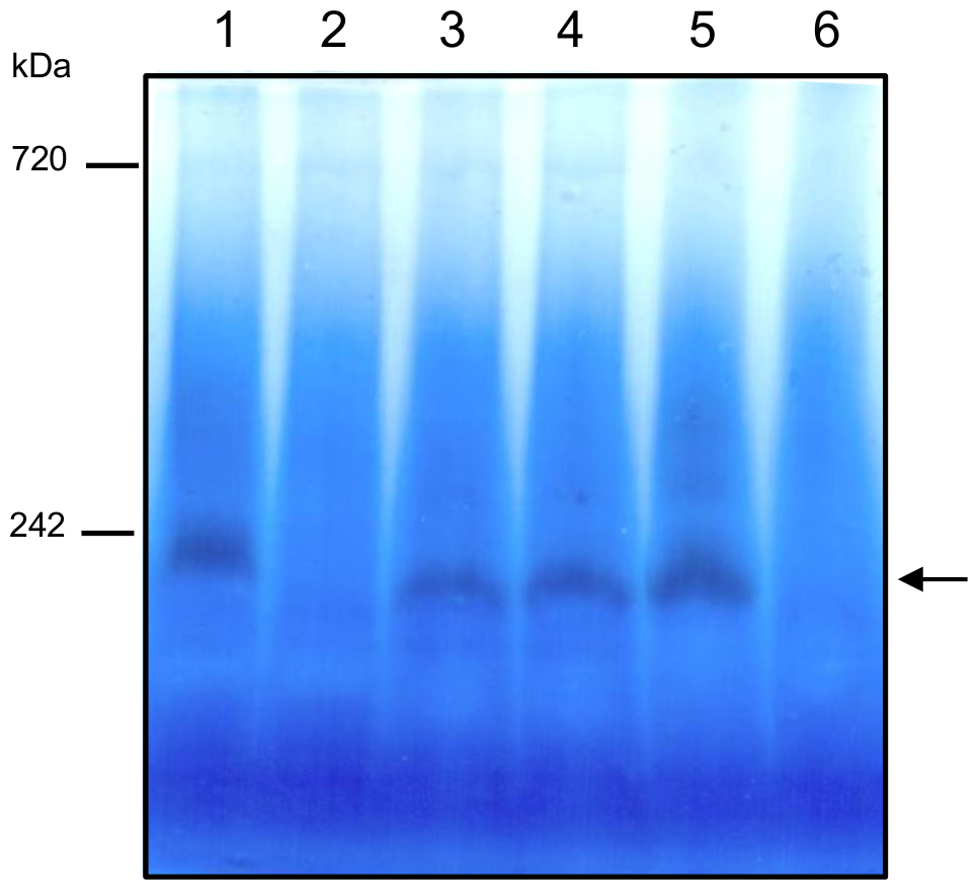
Detection of cytochrome *c* oxidase activity on BN-gel in aerobically grown *S. oneidensis* MR-1 oxidase mutants. Membrane proteins were prepared from wild-type or oxidase mutants *S. oneidensis* MR-1 cells grown aerobically and harvested during the exponential phase of growth. 130 µg of total proteins were loaded on a 5–15% polyacrylamide gel. The band of activity is indicated by an arrow. Lane 1: *S. oneidensis* MR-1. Lane 2: strain SLL01. Lane 3: strain SLL02. Lane 4: strain SLL03. Lane 5: strain SLL05. Lane 6: strain SLL06.

Membrane proteins from *S. oneidensis* MR-1 and oxidase mutant strains grown under aerobic conditions were separated on BN-gel electrophoresis and the cytochrome *c* oxidase activity was revealed (Fig. 5). One band of activity, of molecular mass around 240 kDa, was observed in all strains except for the strain lacking the *cbb*_3_ oxidase (SLL01) and the strain lacking both the *cbb*_3_ and the Cox oxidases (SLL06). If the BN-gel is incubated a longer time for the detection of the cytochrome *c* oxidase activity, a faint band of activity is detected, of molecular mass around 450 kDa (not shown) in all strains except for SLL01 and SLL06. These results strongly suggest that the *cbb*_3_-oxidase is responsible for the bands of activity in the solubilized membranes of the bacterium. Furthermore, the analysis of these bands of activity by ESI-Q-ToF mass spectrometry (data not shown) confirmed that the cytochrome *c* oxidase responsible for in-gel activity is the *cbb*_3_ oxidase since both CcoO and CcoP, the monohemic and dihemic cytochrome *c* subunits of the *cbb*_3_ oxidase respectively are identified. In contrast, none of the A-type Cox subunits was identified. Collectively, these results undoubtedly demonstrate that under aerobic conditions, the cytochrome *c* oxidase activity detected in the membranes from *S. oneidensis* MR-1 arises from the high-affinity *cbb*_3_ oxidase and that, on the contrary, the low-affinity A-type cytochrome *c* oxidase is surprisingly not required for aerobic respiration.

## Discussion

In this report, we investigated the aerobic respiratory pathway in *S. oneidensis* MR-1 to determine the physiological role of the three terminal oxidases in low and in high-O_2_ environments. Our results suggest that the high-affinity *bd*-type oxidase is weakly expressed in aerobic conditions and significantly induced under microaerobic conditions which is consistent with previous studies indicating that *bd* oxidases are typically used by bacteria for aerobic respiration under O_2_-limited conditions [5], [64], [65]. Taken together, our data also reveal that the *cbb*_3_-type oxidase is the most important terminal oxidase under aerobic conditions and has a significant role under microaerobic conditions whereas the low affinity A-type cytochrome *c* oxidase Cox was not detected in the tested conditions even under aerobic conditions. In addition, characterization of the terminal oxidase deletion mutants confirms that the *cbb*_3_-type or the *bd*-type oxidase is required for aerobic growth and that Cox is unexpectedly not involved in aerobic respiration in these experimental conditions.

Our results are overall consistent with those recently reported by Zhou *et al.* [25] who revealed, by measuring the mRNA abundance and the promoter activity in *S. oneidensis* MR-1, that *cbb*_3_-type oxidase and *bd*-type oxidase are important under microaerobic conditions and the *cbb*_3_-type is the major oxidase under aerobic conditions while Cox has no physiological significance. However, it is noteworthy that, in their study, the expression level of the *cco* genes encoding the *cbb*_3_ oxidase was lower under microaerobic conditions than under aerobic conditions and directly proportional to the O_2_ level in aerobic cultures, suggesting that expression of *cco* is favored in O_2_-rich environments. On the contrary, we clearly present evidence that the oxidase activity arising from the *cbb*_3_ oxidase was constant under the tested conditions and thus did not respond to growth conditions. Furthermore, if Zhou *et al.* [25] found that the *cyd* operon was slightly induced at low O_2_ concentration compared to high O_2_ conditions (<5-fold increase in mRNA abundance), the difference observed between aerobic and microaerobic conditions was much more significant at the protein level with a 40-fold increase observed by measuring quinol-dependent oxidase activity. Probably due to translational and/or post-translational regulation mechanisms, these discrepancies emphasize the importance of biochemical analysis at the protein level to understand the global metabolic and regulatory processes in addition to studies of expression at a transcriptional level.

These findings are intriguing since *cbb*_3_ oxidases, given their high affinity for O_2_, are usually repressed at high oxygen tension in many bacteria [66]–[69]. Furthermore, in organisms carrying an *aa*_3_-type and a *cbb*_3_-type oxidase, the low affinity oxidase plays a dominant role under high oxygen conditions whereas the *cbb*_3_ oxidase is induced only under low O_2_ concentrations [67]. In *S. oneidensis* MR-1, the uncommon expression pattern of the cytochrome *c* oxidases is reminiscent of the one found in *Pseudomonas aeruginosa*. In this bacterium, the *cox* genes encoding an *aa*_3_-type oxidase are expressed at a very low level under O_2_-rich growth conditions in LB-medium while the expression level of the genes encoding the *cbb*_3_-1 oxidase was high in the same conditions.

Interestingly, the *cox* genes were found to be significantly induced in *P*. *aeruginosa* under starvation of iron, carbon or nitrogen indicating that the *aa*_3_-type oxidase might be involved in energy conservation under low nutrient conditions [70], [71]. The induction of the *cox* genes under nutrient starvation or other environmental stress conditions should thus be considered in *S. oneidensis* MR-1. Although a functional loss of the A-type Cox oxidase in *S. oneidensis* MR-1, as proposed by Zhou *et al*. [25], cannot be excluded, the presence of the *cox* genes as well as of genes involved in the maturation and assembly of the oxidase in all genome-sequenced *Shewanella* strains leads us to believe that the Cox oxidase has a significant role in some not yet defined conditions. Moreover, in the phylogenetic tree built with the sequences of the catalytic subunits of A, B and C-type oxidases from various Bacteria and Archaea, the A-type Cox from different *Shewanella* species, among them the one from *S. oneidensis* MR-1, clustered with bacterial A-type cytochrome *c* oxidases (data not shown). For *Shewanella* genes, the observed branch lengths did not indicate a high evolutionary rate characteristic of pseudogenes, which is again not in favour of the hypothesis of a functional loss of the A-type Cox.

In addition to the particularity of the membrane terminal oxidase content in *S. oneidensis* MR-1, examination of the derived amino acid sequences emphasized the singularity of the Cox oxidase. Indeed, if sequence analysis allowed to classify Cox in the A1 subfamily and to predict the incorporation of *a*-type heme at least at the low-spin site of CoxA, an uncommon extra C-terminal domain carrying two *c*-type heme binding consensus sequences was identified in the predicted subunit CoxB. Subunit II with an extra domain carrying only one *c*-type heme can be found in bacterial terminal oxidases such as in the *caa*_3_ oxidase from *Thermus thermophilus* or *Bacillus subtilis* [72], [73]. Intriguingly, the C-terminal extension of CoxB from *S. oneidensis* MR-1 likely binds two *c*-type hemes suggesting that this enzyme is a *ccaa*_3_-type HCO. Such an unusual feature was so far described only in *Desulfovibrio* species [74], [75] but we discover that the two *c*-type heme binding motifs were also conserved in some γ-proteobacteria such as *Shewanella* (except in *S. violacea* and *S. benthica* KT99) and some species of the genus *Psychromonas*, *Colwellia* and *Methylosarcina* (data not shown). As in *Desulfovibrio* species, the two heme *c* domains in *S. oneidensis* MR-1 are highly similar to each other (61% sequence identity) suggesting that the diheme domain evolved from a gene duplication event [74].

A study is currently underway to determine experimental conditions under which the *ccaa*_3_-type oxidase Cox would be expressed. It should provide information about the physiological significance of this oxidase in *S. oneidensis* MR-1.

## Supporting Information

Table S1 **Oligonucleotides used in this study.**
(DOCX)
