# Supplementary material for: A Biochemical Approach to Study the Role of the Terminal Oxidases in Aerobic Respiration in Shewanella oneidensis MR-1
Source: PLoS One. 2014 Jan 22;9(1):e86343. doi: 10.1371/journal.pone.0086343 (PMC3899249; doi:10.1371/journal.pone.0086343)
Supplement: Table S1 — Oligonucleotides used in this study. (DOCX) [file pone.0086343.s001.docx]

Table S1**.** Oligonucleotides used in this study.

| **Deleted gene** | **Oligonucleotide** | **Oligonucleotide sequence (5’-3’)** |
| --- | --- | --- |
| **SO2364** | Cbbnew2 | GTAGAAACTAGTGAATAGTCATCTTATCGCCGGTC (SpeI) |
|  | Cbbnew3 | *GCTCAACAACCC*GGCTTAAGGAGCAAACTCGATG |
|  | Cbbnew4 | *GCTCCTTAAGCC*GGGTTGTTGAGCTATGTCAATGC |
|  | Cbbnew5 | ATCGTTGTCGACGTAGCTCGTTTTGTCCACTTGG (SalI) |
| **SO3286** | Bdox2 | GACTAGTAATAAGCCGCCATGGTAGGTCGC (SpeI) |
|  | Bdox3 | *CGCACCACGTAA*GTTGAGAGTAATCTGGTGGGCTC |
|  | Bdox4 | *ATTACTCTCAAC*TTACGTGGTGCGTCCAATTGCTAC |
|  | Bdox5 | ACGCGTCGACGATGGTTACGTGGTTAATTTGCAG (SalI) |
| **SO4607** | Cox2 | GACTAGTAATGCGTGGGGCAATAACACGG (SpeI) |
|  | Cox3 | *AGGTTTTGCATG*CTTAATTTAATCCCCTACTTTCC |
|  | Cox4 | *GATTAAATTAAG*CATGCAAAACCTACCCAAACAACC |
|  | Cox5 | ACGCGTCGACGAAGAAAATCAACGGCAGCTC (SalI) |

Overlapping regions of primers are in italic type. Restriction sites are underlined and restriction enzymes are given in parentheses.
